# Reliability of environmental sampling culture results using the negative binomial intraclass correlation coefficient

**DOI:** 10.1186/2193-1801-3-40

**Published:** 2014-01-22

**Authors:** Sharif S Aly, Jianyang Zhao, Ben Li, Jiming Jiang

**Affiliations:** Veterinary Medicine Teaching and Research Center, School of Veterinary Medicine, University of California, Davis, 18830 Road 112, Tulare, CA 93274 USA; Department of Population Health and Reproduction, School of Veterinary Medicine, University of California, One Shields Avenue, Davis, CA 95616 USA; Department of Statistics, University of California, Davis, CA 95616 USA

**Keywords:** Intraclass correlation coefficient, Generalized linear mixed model, Negative binomial mixed model, Variance components

## Abstract

The Intraclass Correlation Coefficient (ICC) is commonly used to estimate the similarity between quantitative measures obtained from different sources. Overdispersed data is traditionally transformed so that linear mixed model (LMM) based ICC can be estimated. A common transformation used is the natural logarithm. The reliability of environmental sampling of fecal slurry on freestall pens has been estimated for *Mycobacterium avium* subsp. *paratuberculosis* using the natural logarithm transformed culture results. Recently, the negative binomial ICC was defined based on a generalized linear mixed model for negative binomial distributed data. The current study reports on the negative binomial ICC estimate which includes fixed effects using culture results of environmental samples. Simulations using a wide variety of inputs and negative binomial distribution parameters (r; p) showed better performance of the new negative binomial ICC compared to the ICC based on LMM even when negative binomial data was logarithm, and square root transformed. A second comparison that targeted a wider range of ICC values showed that the mean of estimated ICC closely approximated the true ICC.

## Introduction

In the simple case of estimating the correlation among 2 factors with a set of quantitative observations, an investigator may elect to utilize the Spearman Rank correlation coefficient or Pearson’s correlation coefficient assuming the observations are independent. The measure of agreement κ can be estimated for correlation between binary observations. For more complex data structures that may include either crossed or nested factors of a latent character, the investigator may utilize the Intraclass Correlation Coefficient (ICC). The ICC is related to unexplained variance at the subject level. More specifically, the ICC is defined as the ratio of the covariance of measurements from the factor of interest to the marginal variance of the observations. Ranging between 0 and 1, an ICC close to 1 indicates that the difference in observations due to the factor of interest are ignorable. Hence, using variance estimates attributable to each of a study’s factors, the ICC can be used as a measure of similarity in observations between subjects due to a particular factor. A direct application of the ICC is a measure of the correlation between subjects in a reliability and repeatability gauge study (Aly et al. 2009; Kittawornrat et al. 2012).

Investigators analyze and obtain variance estimates for normally distributed data using linear mixed models (LMM) or non-normally distributed data using generalized linear mixed models (GLMM). Health science researchers more commonly work with count data and while the ICC for the LMM has been extended to the Poisson case (Carrasco and Jover 2005), its equivalence for count data with overdispersion was only recently described (Carrasco 2010). Until the ICC for negative binomial distributed data was developed, researchers transformed such data using different transformations to make their data normally distributed in order to use LMM and their ICC.

An example of count data that may commonly be overdispersed is bacterial culture results. Culture results are commonly reported as colony forming units per specimen mass or culture medium tube. Another example is parasite counts which are commonly reported as parasitic stage count per gram of specimen. Given the nature of such infectious agents, they can exist in very large numbers within their hosts, at the same time not all potential hosts in a population are infected. In fact, more hosts tend to be uninfected leading to the inequality of the mean and variance of the data, hence overdispersion. In the current study, we report on a reliability analysis for environmental sampling to quantify *Mycobacterium avium* subspecies *paratuberculosis* (MAP) on California free-stall dairies (Aly et al. 2009). A previous study with these data was unique in that it involved the use of nested and crossed factors and used the natural logarithm to attain normally distributed data for a LMM analysis and ICC estimation. Such transformations may normalize the data provided the number of replicates was large and the variance components were small (Solomon and Taylor 1999). Both sample size and magnitude of variance conditions may be difficult to attain with negative binomial distributed data especially when replicates are limited due to cost or subject use limitations such as in health sciences research. The performance of the negative binomial ICC has not been compared to LMM ICC using previously described data transformations in multilevel models with crossed and nested random effects.

Hence, the objectives of this study were to specify a negative binomial mixed model and estimate and contrast the performance of the resulting ICC to that based on estimates from linear mixed models of several data transformations. In addition to the reliability study on environmental sampling to quantify MAP in dairy pens, a wide variety of negative binomial distributed data was simulated to contrast estimator performance.

## Methods

### ICC for the negative binomial mixed model

For the purpose of deriving the ICC that estimates the similarity of samples collected by two veterinarians on the same day from the same pen. Here *y*_*ijkl*_ denotes the observed value of the *j*^*th*^ pen in *i*^*th*^ dairy, the *k*^*th*^ day, and the *l*^*th*^ veterinarian (*i* = 1, 2,..*m*; *j* = 1, 2,..*n*_m_; *k* = 1, 2,..*s*; *l* = 1, 2,..*t*); the total number of observations is *N* = *st*∑*n*_*m*_. We assume that the conditional distribution of *y*_*ijkl*_ given the random effects *a*, *b*, *c*, *d* (dairy, pen, day and veterinarian, respectively) is distributed negative binomial with the number of successes needed *r* and probability of success *p*_*ijkl*_, or *NB(r; p*_*ijkl*_*)*. In this distribution, *r* is fixed for all *y*_*ijkl*_. Furthermore, it is assumed that , where *μ*_*ijkl*_ is the conditional expectation of *y*_*ijkl*_ given *p*_*ijkl*_. Recall  (Casella and Berger 2002), where *a*_i_ (*i* = 1, 2,…,*m*) are independent and distributed as N(0; ), *b*_*i*_ (*i* = 1,2,…*n*_*m*_) are independent and distributed as N(0; ), *c*_*i*_ (*i* = 1,2,..*s*) are independent and distributed as N(0; ), and *d*_i_ (*i* = 1, 2,..*t*) are independent and distributed as N(0; ). Hence the conditional expectation

and the conditional probability

thereby the conditional distribution of *y*_*ijkl*_ is NB(r, )which is a special case of the GLMM. The ICC for the similarity in Herrold’s egg yolk medium (HEYM) culture results for MAP in samples collected by 2 different collectors (*l*_*1*_ and *l*_*2*_) will be derived as an example. Given the model assumptions and study design,  and  are conditionally independent if *l*_*1*_ ≠ *l*_*2*_, so the conditional expectation of their product is the product of their conditional expectations. Therefore,

The random variable  has the distribution N , hence  has the distribution log-normal .

According to the expectation of the log-normal distribution, we have:

Similarly,

The covariance between two measurements that are generated by different veterinarians but otherwise are identical in all factors is the difference between the expectation of their product and the product of their expectations, that is:

On the other hand, according to the variance of the log-normal distribution,

Hence, the expectation of conditional variance of the observations can be expressed as:

Therefore, the variance of the observations is:

It follows then that the ICC for the negative binomial mixed model for the similarity between samples collected by two different veterinarians on the same day and from the same pen is:

When the variance of data is much larger than its expectation, the negative binomial distribution is often used as an alternative to the Poisson distribution. The random effects follow the normal distribution and the link function is the logarithm. Based on this formula, the ICC is no longer just based on the random effects, but also the fixed effect intercept and the number of successes. Thus, the negative binomial mixed model may be more reasonable than the LMM or the Poisson GLMM when count data are overdispersed.

### Simulations

Simulations were used to compare the performance of the new negative binomial ICC to that estimated after traditionally transforming count data to normalize it using transformation such as the logarithm, square root, square, or their inverse values (Carrasco and Jover 2005). To compare the performance of the ICC estimator derived for the negative binomial GLMM to the ICC used in traditional methods such as LMM of normalized count data, 16 scenarios were generated. Fixed estimates of input parameters were used in each of the 16 scenarios and their respective true ICC as summarized in Table 1. The scenarios included 2 different estimates of r (r = 1 and r = 2), numbers of successes. In addition, zero and non-zero intercepts (β =0 and β =2), 2 different between-dairy variance estimates (0.5 and 1), and 2 different between-veterinarian variance estimates (0.1 and 0.5) were assumed. The justification behind the use of fixed estimates for the between-pen and between-day variances is that by equation (1), these variances influence the ICC in the same way, as the between-dairy variance; therefore it is reasonable to vary only one of them. Based on the study by Aly et al. (2009) there were 4 factor levels: dairy, pen, veterinarian and day. Pens were nested within dairy. In dairy *i* , *i* = 1, 2, 3, 4 has *j* pens; where for *i* = 1, *j* = 1,…, 8; for *i* = 2, *j* = 1,…, 11; for *i* = 3, *j* = 1,…, 7; and for *i* = 4, *j* = 1,…, 4. Pens were cross-classified by veterinarian *l*; *l* = 1, 2; and day *k*; *k* = 1, 2, 3. Data were generated under the assumption of negative binomial GLMM with log link using all four factors *a*, *b*, *c*, *d* included as random effects. Each sample dataset consisted of 180 observations. Each simulation followed the following procedure:

1. Randomly generate normal random effects *a*_*i*_, *b*_*ij*_, *c*_*k*_, *d*_*l*_(*i* = 1, 2,.. *n*_*m*_; *k* = 1, 2.. *s*; *l* = 1, 2,.. *t*) with respective scenario’s variances
2. Sum the intercept and random effects as conditional expectation , *β* is estimated intercept from field data
3. Randomly generate negative binomial variable *Y*_*ijkl*_ ~ *NB*(*r*, *μ*_*ijkl*_) r is number of successes
4. Estimate model parameters: intercept *β*, number of successes *r* and random effects
5. Calculate the ICC

**Table 1:** **Parameters of a simulation to compare the true and estimated negative binomial Intraclass Correlation Coefficient (ICC) using an example of culture results for a specific bacterium in pen floor samples (variance 0.5) collected over several days apart and simultaneously by different veterinarians and across different dairies**

Scenario	r	β	Variance				E(Y)	True ICC
			Dairy	Pen	Day	Veterinarian		
1	1	0	0.5	0.5	0.2	0.1	1.92	0.3382
2	1	0	1	0.5	0.2	0.1	2.46	0.3888
3	1	2	0.5	0.5	0.2	0.1	14.15	0.362
4	1	2	1	0.5	0.2	0.1	18.17	0.4011
5	2	0	0.5	0.5	0.2	0.1	1.92	0.4616
6	2	0	1	0.5	0.2	0.1	2.46	0.5275
7	2	2	0.5	0.5	0.2	0.1	14.15	0.5072
8	2	2	1	0.5	0.2	0.1	18.17	0.5503
9	1	0	0.5	0.5	0.2	0.5	2.34	0.2236
10	1	0	1	0.5	0.2	0.5	3	0.2574
11	1	2	0.5	0.5	0.2	0.5	17.29	0.2319
12	1	2	1	0.5	0.2	0.5	22.2	0.2617
13	2	0	0.5	0.5	0.2	0.5	2.34	0.3037
14	2	0	1	0.5	0.2	0.5	3	0.3476
15	2	2	0.5	0.5	0.2	0.5	17.29	0.3192
16	2	2	1	0.5	0.2	0.5	22.2	0.3556

One hundred simulated data sets were generated under each scenario. For each simulated data set, the ICC was estimated using four different methods: 1) the negative binomial GLMM, 2) LMM of raw data (untransformed); 3) LMM of square root transformed data; and 4) LMM of logarithm transformed data where taking logarithm of zero was avoided by replacing zeros with 0.5. For LMM, restricted maximum-likelihood estimation (REML) was used, while maximum-likelihood (ML) estimation was used for the GLMM. Relative bias, variance of the ICC, and mean square error (MSE) of the ICC estimate were calculated to evaluate the performance of the ICC. The relative bias was calculated as the difference between the mean of estimated ICC and it’s true value, variance was calculated by unbiased estimation based on the simulation, and MSE was calculated as the sum of squared bias and variance.

A second simulation explored the performance of the ICC estimate over a wider range. The mean estimated ICC was computed using 400 simulations per combination of number of successes (r = 5 and r = 30) and variance estimates for dairy and veterinarian (0 to 1 in increments of 0.2).

### Field data analysis

Finally, field data used in the report by Aly et al. (2009) were analyzed using the negative binomial GLMM. Briefly, environmental samples were collected every other day on 3 different occasions from 4 California dairies between November 2006 and June 2007. Samples were cultured using bacterium-specific medium using standard microbiological procedures as reported by Aly et al. (2009). Confidence intervals for model parameters were obtained based on parameter estimates from the field data and using parametric bootstrap similar to that described in Table 1 (Efron and Tibshirani 1993). The resulting negative binomial based ICC was contrasted to that estimated from transformed data and reported previously by Aly et al. (2009). The R package lme4 was used for LMM analysis, and the package glmmADMB for GLMM analysis. All packages were loaded in the R 2.15.1 environment.

## Results

Results of the first simulation targeted a range of ICC values based on 16 combinations of input parameters (r, β, variances of dairy, pen, veterinarian and day) and are presented in Table 2. The relative bias in the ICC, variance and MSE were compared for the ICC estimates based on the negative binomial model to those based on the LMM of raw (untransformed) and transformed data. The negative binomial model ICC had the least absolute relative bias in 5 of the 16 scenarios (3, 4, 5, 6 and 8) that were characterized by small variance estimates for veterinarian (0.1). In comparison, the ICC based on LMM of raw data had the most number of scenarios with the least absolute relative bias (9, 10, 11, 12, 14, 15 and 16) characterized by large variance estimates for veterinarian (0.5). In terms of variance, the negative binomial ICC had the most number of scenarios with the least variance (1 to 5, 7, 8, 11, 12, 15). Similarly for MSE, the ICC based on the negative binomial model had the least MSE in 11 of the 16 scenarios (1 to 8, 11, 12, 15 and 16).

**Table 2:** **Point estimate (PE) relative bias, variance, and mean square error (MSE) of Intraclass Correlation Coefficient (ICC) for culture results of samples collected by 2 veterinarians and based on the negative binomial mixed model, linear mixed model with raw data, square-root transformed data and log-transformed data (bold values are nearest to zero within a row)**

Scenario	Parameter	Negative binomial	Transformed data		
			Raw	Natural logarithm	Square root
1	PE relative bias%	-10.35	-16.14	-5.41	**-5.32**
	Variance	**0.0098**	0.0138	0.0145	0.0137
	MSE	**0.011**	0.0168	0.0148	0.014
2	PE relative bias%	-10.8	-17.21	**-1.13**	-2.55
	Variance	**0.0108**	0.0136	0.0198	0.0183
	MSE	**0.0126**	0.0181	0.0198	0.0184
3	PE relative bias%	**-5.33**	-7.65	8.45	12.43
	Variance	**0.0052**	0.0118	0.0115	0.012
	MSE	**0.0056**	0.0126	0.0124	0.014
4	PE relative bias%	**-9.3**	-16.93	9.75	9.7
	Variance	**0.0067**	0.0107	0.0205	0.0152
	MSE	**0.0081**	0.0153	0.022	0.0167
5	PE relative bias%	**-8.28**	-18.37	-10.46	-10.92
	Variance	**0.0114**	0.0135	0.0133	0.0136
	MSE	**0.0129**	0.0207	0.0156	0.0161
6	PE relative bias%	**-19.51**	-30.33	-21.06	-22.33
	Variance	0.0148	**0.0138**	0.0162	0.0161
	MSE	**0.0254**	0.0394	0.0285	0.03
7	PE relative bias%	-8.02	-14.27	**0.41**	2.54
	Variance	**0.0095**	0.012	0.0158	0.0122
	MSE	**0.0112**	0.0172	0.0158	0.0124
8	PE relative bias%	**-5.89**	-15.66	9.03	7.11
	Variance	**0.009**	0.0107	0.016	0.0131
	MSE	**0.01**	0.0181	0.0185	0.0146
9	PE relative bias%	17.53	**3.26**	27.01	26.74
	Variance	0.0129	**0.0105**	0.0126	0.0118
	MSE	0.0144	**0.0106**	0.0162	0.0154
10	PE relative bias%	8.55	**-0.51**	31.12	27.35
	Variance	0.0165	**0.0145**	0.025	0.0225
	MSE	0.017	**0.0145**	0.0314	0.0275
11	PE relative bias%	30.36	**27.17**	62.05	66.58
	Variance	**0.0104**	0.0134	0.0144	0.015
	MSE	**0.0154**	0.0174	0.0351	0.0388
12	PE relative bias%	19.56	**16.28**	57.01	55.29
	Variance	**0.0118**	0.0157	0.0193	0.0183
	MSE	**0.0144**	0.0175	0.0416	0.0392
13	PE relative bias%	13.24	**7.84**	18.41	18.51
	Variance	0.0213	0.0185	**0.0176**	0.0183
	MSE	0.0229	**0.0191**	0.0207	0.0215
14	PE relative bias%	7.22	**-2.79**	17.15	15.39
	Variance	0.0217	**0.0172**	0.027	0.0255
	MSE	0.0223	**0.0173**	0.0306	0.0284
15	PE relative bias%	28.41	**23.59**	45.99	48.25
	Variance	**0.0154**	0.0182	0.0178	0.0188
	MSE	**0.0236**	0.0239	0.0394	0.0425
16	PE relative bias%	22.69	**19.99**	51.97	50
	Variance	0.0216	**0.0205**	0.0262	0.023
	MSE	0.0281	**0.0256**	0.0604	0.0546

**Figure 1:**
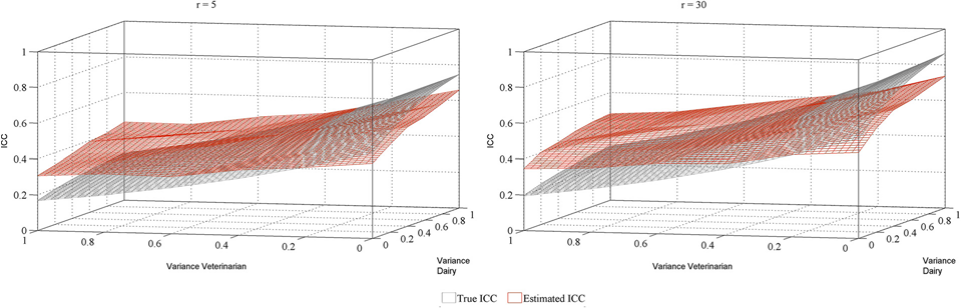
**Performance of the Intraclass Correlation Coefficient (ICC) from a negative binomial mixed model with the number of successes** ***r*** **= 5 and =30.** The data simulated were for the example of culture results for a specific bacterium in pen floor samples (variance 0.5) collected over 3 days 24 hours apart (variance 0.2) and simultaneously by 2 different veterinarians across 4 dairies (0 to 1 in increments of 0.2).

The second simulation performed to investigate the effect of larger number of successes (r = 5 and r = 30) and a wider range of variance estimates for dairy and veterinarian that also include zero. Figure 1 showed that the mean of the estimated ICC and the true ICC were similar as estimates of variance due to veterinarian ranged from 0.1 to 0.3 even as variance due to dairy increased to 1. However, as depicted in the diverging planes, the difference between the estimated and true ICCs increased towards extreme variance estimates. Both behaviors were consistent in a higher number of successes (r = 30). Figure 1 depicts the differences between the true ICC and the mean of the respective estimated ICC based on simulations.

Results of the negative binomial GLMM are summarized in Table 3. The negative binomial ICC was estimated to be 0.5207 (95% CI = 0.4033, 0.6091) compared to the estimate based on natural log transformed data which was 0.6730 (95% CI = 0.5130, 0.8340).

**Table 3:** **Parameter estimates from a negative binomial generalized linear mixed model for culture results from a study on the reliability of an environmental sampling protocol and the Intraclass Correlation Coefficient (ICC) for similarity in samples collected by two veterinarians on the same day and from the same pen**

		95% Confidence interval	
Parameter	Estimate	Lower	Upper
β	1.9516	1.3745	2.6011
r	1.379	1.0138	2.0225
σ_a_	0.2691	2.07E-09	0.8657
σ_b_	1.352	0.5786	2.028
σ_c_	2.11E-09	2.06E-09	0.0303
σ_d_	4.72E-04	2.06E-09	0.0359
ICC	0.5207	0.4033	0.6091

^a^random effect for dairy *i*, *i* = 1, 2, 3, 4.

^b^random effect for pen *j*, where for *i* = 1, *j* = 1,…,8, for *i* = 2, *j* = 1,…,11, for *i* = 3, j = 1,…,7 and for *i* = 4, *j* = 1,…,4.

^c^random effect for day *k* of sample collection, where *k* = 1,2,3.

^d^random effect for collector *l*, where *l* = 1, 2 and day k; k = 1, 2, 3.

## Discussion

The current study updates an earlier report on the reliability of environmental sampling to quantify MAP in freestall dairy pens utilizing the negative binomial ICC for count data. A unique character of the negative binomial ICC is the inclusion of the fixed effect intercept estimate unlike the ICC based on LMM which is based soley on variance components. Fixed effects are similarly included in the formula for the poisson ICC however the negative binomial ICC also includes *r*, the distribution parameter for number of successes. A performance comparison of the ICC estimates showed that the negative binomial ICC was more suitable for count data that is overdispersed given the smaller MSE and variance estimate than the ICC from LMM. Relative bias tended to the least in more scenarios (7 out of 16) with LMM compared to the GLMM based ICC. The lower relative bias with LMM may be explained by the use of REML estimation. The choice of MLE for GLMM was justified by that REML for GLMM has not been well defined, unlike for LMM (Jiang 2007). Nevertheless, the ICC for the negative binomial data outperformed that based on LMM of logarithm or square root transformed data with respect to MSE and variance. Results of a second simulation with highly overdispersed data showed that the NB ICC tended to overestimate the true ICC with higher variance components and under estimate with lower variance components. This expected behavior was consistent in a higher number of successes (r = 30) which confirms stability of the estimator over a wide variety of negative binomial distributed data.

Aly et al. (2009) estimated the ICC for similarity in HEYM culture results of MAP in samples collected by two different collectors on the same day and from the same pen to be 67.3%. The current study showed that the similarity in culture results estimated using the negative binomial ICC could be as low as 52.07%. Such a difference is expected given that the culture results are overdispersed count data. One reason for overdispersion may relate to the culture of MAP on HEYM protocol itself. Specifically fecal slurry samples undergo a decontamination step to limit bacterial growth on HEYM to mycobacteria. The decontamination step also reduces the number of MAP organisms resulting in samples with low MAP counts which may test negative (zero colony forming units) increasing the variance. For this reason, quantitative real-time PCR (qPCR) may remain the most suitable choice for testing freestall pen environmental samples for MAP.
